# Supplementary material for: A co-designed website (FindWays) to improve mental health literacy of parents of children with mental health problems: Protocol for a pilot randomised controlled trial
Source: PLoS One. 2023 Mar 21;18(3):e0273755. doi: 10.1371/journal.pone.0273755 (PMC10030009; doi:10.1371/journal.pone.0273755)
Supplement: S2 File — (PDF) [file pone.0273755.s002.pdf]

Parent / Guardian Information Statement

**HREC Project Number:**	**HREC 75854**
**Research Project Title:**	FindWays: testing a co-designed website for parents to find ways to help their child's behaviour or emotions.
**Principal Researcher:**	Dr Daniel Peyton is a paediatrician (children's doctor) and PhD student from the Murdoch Children's Research Institute and the University of Melbourne.
**Version Number:**	1.4
**Version Date:**	*05/04/22*

Thank you for taking the time to read this **Parent/Guardian Information Statement**. We would like to invite you to participate in a research project that is explained below.

This document is 6 pages long. Please make sure you have all the pages.

What is an Information Statement?

An Information Statement tells you about the research project. It clearly explains exactly what the research project will involve. This information is to help you decide whether or not you would like to take part in the research. Please read it carefully.

Before you decide if you want to take part or not, you can ask us any questions you have about the project. You may want to talk about the project with your family, friends, or health care worker.

Taking part in the research is up to you

It is your choice whether or not you can take part in the research. You do not have to agree if you do not want to. If you decide you do not want to take part, it will not affect the treatment and care your child gets at [paediatric clinic name].

1. What is the research project about?

We know that families can face long wait times to see a pediatrician for a behavioral or emotional issues. In the meantime, families can find it hard to know what to do and where else to find help. To help parents while they wait to see the paediatrician, Dr Peyton has created a new website, designed with parents in Geelong. This website is designed to be used on your phone.

Dr Peyton wants to know whether this new, co-designed website will help families to manage their child's emotions or behaviors while they wait to see a paediatrician. This pilot trial will involve around 60 parents. It will help us understand whether parents want to use this new website, whether they can use, and whether we should make the website available to more families outside of Geelong.

2. Who is funding this research project?

Daniel Peyton is being funded by Barwon Health and National Health and Medical Research Council (APP1189935). The project is supported by Charity Drive Days and the Murdoch Children's Research Institute.

3. Why am I being asked to take part?

We are asking you to take part because:

- Your child was referred to a paediatrician with a behavioural or emotional issue
- Your child is between 2 and 12 years old.
- Your child was referred to one of three private paediatric clinics in Geelong.

4. What do I need to do in this research project?

This research project will last 4 months. You will be asked to fill out an online survey twice – once . at the start of the project, and again 4 months later. The survey will last about 20 minutes. It will ask you questions about how old your child is, how many people live with you, your education and employment, as well ask questions about your child's behaviour and emotions, and your own emotions. We will also ask you some questions about what services you may have used in the past to help your child.

We will put you into one of two groups: a website group and a no website group (also called a control group). This will be done by chance, similar to tossing a coin. You have an equal chance of being in either group.

This section gives you more information about the two groups.

a. Website group

In this group you will be given access to a new website called FindWays. This website was designed with parents in Geelong to help parents find ways to help their child's behaviour or emotions while waiting to see a paediatrician. Only you will be sent a link to the website. You can share this link with your partner, but not to friends or family outside of your household. Please don't share this website publicly or on social media like facebook or twitter. This is so we can first work out what works and doesn't work with the website before we can make it widely available to other parents in Geelong.

If you are a parent in this group, we will also send you five reminders over four months about the website. These reminders are in the form of text message and email prompts. They will give you your unique link to the website and a short message about how the website might be able to help your child. You can ask to stop these reminders at any time. If you do not want to use the website, this will not affect your participation into this project or your relationship with [paediatric clinic name] or the research team involved in this project.

Optional Consent

At the end of the 4 months, we will ask you to participate in a 30-minute phone interview. This interview will help us get a better understanding of your experience using the website. Such as what you liked about it or didn't like about it. We don't need to talk to your child during this interview. We will make a digital audio-recording of the interview so we can concentrate fully on listening to what you have to say. This audio recording will be stored securely on a MCRI password protected computer. After the interview, either Dr Peyton or a transcription company used by MCRI called Outscribe, will transcribe the recording. This means we will make a full written copy of the recording.

We will securely store a copy of the audio recording on MCRI servers for 5 years, then they will be deleted. The audio recording will not be de-identified.

We would like you to consider letting us contact you about new research projects that may be suitable for you. We would contact you with full details about any new project. It is your choice whether you or your child takes part in these projects. You can say no to them if you want to.

b. No website group (control group)

In this group you will be receive standard care. This means you and your child can still access all your regular supports but you will not have access to the website.

5. Can I stop taking part in this project?

You can stop taking part in the project at any time. You just need to tell us so. You do not need to tell us the reason why. If you stop the project, we will use any information already collected.

6. What are the possible benefits for my child and other people in the future?

We cannot guarantee that you or your child will get any benefits from this project. However, there is a chance that the website will help you and your child by giving you new information about strategies or local services that could help your child's behaviour or emotions. The information you give us from this project, will help us know which parts of the website are useful and how we might improve the website to be better support other families who are trying to get help for their child's behaviour or emptions.

7. What are the possible risks, side-effects, discomforts and/or inconveniences?

We have tried to make sure that the questions in the survey, the questions in the optional phone interview, and the information on the website are not distressful. However, if you are worried by any of the questions, you do not need to answer them.

The interviewer will not be able to provide you with any advice about your child. But if the interview makes you feel worried, the interviewer will be able to suggest ways of getting assistance.

8. What will be done to make sure my and my child's information is confidential?

In this project we will collect and use personal and health information about you (including about how you use and navigate the website if you are part of the website group) and your child for research purposes. Any identifying information will be treated as confidential. It will be used only in this project. We can disclose the information only with your permission, or that of your child if they are able to do so, except as required by law.

All information will be stored securely at the Murdoch Children's Research Institute.

The following people may access information collected as part of this research project:

- the research team involved with this project
- The Royal Children's Hospital Human Research Ethics Committee

The stored information will be re-identifiable. This means that we will remove identifying information such as your name and give the information a special code number. Only the research team can match your name to the code number, if it is necessary to do so.

The information collected from parents will be kept for five years.

Please read our Privacy Policy available at: https://www.mcri.edu.au/privacy-policy for more information about how you may access and correct your personal information and how to complain about a breach of the Privacy Act and how Murdoch Children's Research Institute will deal with such a complaint.

You have the right to access and correct the information we collect and store you. This is in accordance with relevant Australian and/or Victorian privacy and other relevant laws. Please contact us if you would like to access this information.

At the end of the project, we may present the results at conferences. We may also publish the results in medical journals. This will be done in such a way that you or your child cannot be identified.

The results of this research will be used by Dr Daniel Peyton, for his PhD.

9. Will we be informed of the results when the research project is finished?

We will send you a summary of the overall project results. The summary will be of the whole group of project participants, not your individual results.

10. Who should I contact for more information?

If you would like more information about the project, please contact:

**Name:**	Daniel Peyton
**Contact telephone:**	0432 706 921
**Email:**	findways@mcri.edu.au

If you need emergency support, you can call:

- 000
- Child and adolescent mental health service on 1300 094 187
- Lifeline 13 11 14
- Kid's Help Line 1800 55 1800

You can contact the Director of Research Ethics & Governance at The Royal Children's Hospital Melbourne if you:

- have any concerns or complaints about the project
- are worried about your child's rights as a research participant
- would like to speak to someone independent of the project.

The Director can be contacted by telephone on (03) 9345 5044.
