# Supplementary material for: A co-designed website (FindWays) to improve mental health literacy of parents of children with mental health problems: Protocol for a pilot randomised controlled trial
Source: PLoS One. 2023 Mar 21;18(3):e0273755. doi: 10.1371/journal.pone.0273755 (PMC10030009; doi:10.1371/journal.pone.0273755)
Supplement: S3 File — (DOCX) [file pone.0273755.s003.docx]

| protocol  **HREC 75854** |
| --- |
| **FindWays: testing a co-designed website for parents to find ways to help their child’s behaviour or emotions.**  **Short title: FindWays Pilot** |
| Protocol number: HREC 75854  Protocol Version # and date: V1.4.2 17/02/23  **Document history:**   \| **Version Number and Date** \| **Summary of changes** \| \| --- \| --- \| \| **V1.0**  **18/05/21** \| Initial release of protocol \| \| **V 1.1**  **25/05/21** \| After review by GW, NH and HH. \| \| **V1.2**  **08/06/21** \| Post peer review with the following changes:   - Changes to telephone script screening to ask about prior autism diagnosis - Removal of the question regarding unmet needs on the parent facing survey (including objectives and measures section of the protocol). - Inclusion of research mobile phone number to participant information statement and emergency contact for participant distress. \| \| **V1.3**  **03/03/22** \| Post review by HREC. CI Peyton has made changes to the protocol and PIS based on HREC’s comments dated 2/7/21. \| \| **V1.4**  **05/04/22** \| Response to ethics comments   1. Changes to PIS emergency contact 2. Changes to PIS for holding of audio details \| \| **V1.4.2**  **17/02/23** \| We are making an administrative change to the protocol to facilitate publication with PLOS One. PLOS One requires publication of the ethically approved protocol online, alongside the submitted manuscript. In order to publish the ethics protocol online, we need to remove the below confidentially paragraph, appendices and a figure. \| |
| **Statement of Compliance**  This clinical trial will be conducted in compliance with all stipulation of this protocol, the conditions of the ethics committee approval, the NHMRC National Statement on ethical Conduct in Human Research (2007 and all updates), the Integrated Addendum to ICH E6 (R1): Guideline for Good Clinical Practice E6 (R2), dated 9 November 2016 annotated with TGA comments and the NHMRC guidance Safety monitoring and reporting in clinical trials involving therapeutic goods (EH59, 2016). |

Contents

[PROTOCOL SYNOPSIS 5](#_Toc127358286)

[GLOSSARY OF ABBREVIATIONS 8](#_Toc127358287)

[INVESTIGATOR AGREEMENT 9](#_Toc127358288)

[1. ADMINISTRATIVE INFORMATION 10](#_Toc127358289)

[1.1. Trial registration 10](#_Toc127358290)

[1.1.1. Trial registry 10](#_Toc127358291)

[1.2. Sponsor 10](#_Toc127358292)

[1.3. Expected duration of study 10](#_Toc127358293)

[1.4. Contributorship 10](#_Toc127358294)

[1.5. Stakeholder involvement 11](#_Toc127358295)

[2. INTRODUCTION AND BACKGROUND 12](#_Toc127358296)

[2.1. Trial rationale and aim 12](#_Toc127358297)

[2.2. Background 12](#_Toc127358298)

[2.3. Risk/Benefit assessment 15](#_Toc127358299)

[2.3.1. Known potential risks 15](#_Toc127358300)

[2.3.2. Known potential benefits 16](#_Toc127358301)

[2.3.3. Assessment of potential risks and benefits 17](#_Toc127358302)

[3 TRIAL OBJECTIVES AND OUTCOMES 18](#_Toc127358303)

[3.1 Objectives 18](#_Toc127358304)

[3.1.1 Primary objective 18](#_Toc127358305)

[3.1.2 Secondary objectives 18](#_Toc127358306)

[4 TRIAL DESIGN 24](#_Toc127358307)

[4.1 Overall design 24](#_Toc127358308)

[4.2 Trial population 24](#_Toc127358309)

[4.3 Eligibility criteria 25](#_Toc127358310)

[4.3.1 Inclusion criteria 26](#_Toc127358311)

[4.3.2 Exclusion criteria 26](#_Toc127358312)

[4.4 Screen failures 26](#_Toc127358313)

[4.5 Recruitment and identification of potential participants 26](#_Toc127358314)

[4.6 Consent 29](#_Toc127358315)

[5 INTERVENTION 29](#_Toc127358316)

[5.1 Intervention arms and the intervention 29](#_Toc127358317)

[5.2 Intervention(s) 30](#_Toc127358318)

[5.2.1 Measurement of participant compliance 30](#_Toc127358319)

[5.2.2 Discontinuation from trial intervention 31](#_Toc127358320)

[6 RANDOMISATION AND BLINDING 31](#_Toc127358321)

[6.1 Randomisation and concealment mechanism 31](#_Toc127358322)

[6.2 Breaking of the trial blind 31](#_Toc127358323)

[6.2.1 On trial 31](#_Toc127358324)

[7 TRIAL VISITS AND PROCEDURES 32](#_Toc127358325)

[7.1 Trial timeline 32](#_Toc127358326)

[7.2 Schedule of assessments 33](#_Toc127358327)

[7.3 Description of procedures 34](#_Toc127358328)

[7.3.1 Withdrawal of consent - participant withdraws from all trial participation 35](#_Toc127358329)

[7.3.2 Losses to follow-up 36](#_Toc127358330)

[7.3.3 Replacements 36](#_Toc127358331)

[7.3.4 Trial Closure 36](#_Toc127358332)

[7.3.5 Continuation of therapy 36](#_Toc127358333)

[8 SAFETY MONITORING AND REPORTING 36](#_Toc127358334)

[8.1 Reporting of safety events 40](#_Toc127358335)

[9 DATA AND INFORMATION MANAGEMENT 40](#_Toc127358336)

[9.1 Overview 40](#_Toc127358337)

[9.2 DATA MANAGEMENT 41](#_Toc127358338)

[9.2.1 Data generation (source data) 41](#_Toc127358339)

[9.2.2 Data capture methods and data use, storage, access and disclosure during the trial 41](#_Toc127358340)

[9.2.3 Data confidentiality 42](#_Toc127358341)

[9.2.4 Quality assurance 43](#_Toc127358342)

[9.2.5 Record Retention 43](#_Toc127358343)

[10 TRIAL OVERSIGHT 43](#_Toc127358344)

[10.1 Governance structure 43](#_Toc127358345)

[11 STATISTICAL METHODS 44](#_Toc127358346)

[11.1 Sample Size Estimation 44](#_Toc127358347)

[12 ETHICS AND DISSEMINATION 44](#_Toc127358348)

[12.1 Research Ethics Approval & Local Governance Authorisation 44](#_Toc127358349)

[12.2 Amendments to the protocol 44](#_Toc127358350)

[12.3 Protocol Deviations and Serious Breaches 45](#_Toc127358351)

[13 CONFIDENTIALITY 45](#_Toc127358352)

[14 PARTICIPANT REIMBURSEMENT 45](#_Toc127358353)

[15 FINANCIAL DISCLOSURE AND CONFLICTS OF INTEREST 45](#_Toc127358354)

[16 DISSEMINATION AND TRANSLATION PLAN 45](#_Toc127358355)

[17 REFERENCES 46](#_Toc127358356)

[17.1 Appendix 1: Division of sponsor responsibilities between sponsor and sponsor-investigator 48](#_Toc127358357)

# PROTOCOL SYNOPSIS

| ***TITLE*** | FindWays: testing a co-designed website for parents to find ways to help their child’s behaviour or emotions. |
| --- | --- |
| ***Short Title*** | FindWays Pilot |
| ***TRIAL description*** | FindWays Pilot is a pilot RCT of a new co-designed website, FindWays. FindWays is for parents of children with behavioural or emotional problems aged 2-12 years who have been referred to see a paediatrician. It is designed to provide parents with information and resources to support their child while they wait to see a paediatrician. |
| ***Objectives*** | The primary objective of this study is to assess the acceptability and feasibility of:   1. a co-designed website (FindWays) for parents of children aged 2-12 years old with behavioural and/or emotional problems referred to a paediatrician for their first appointment; and 2. the study design and measures, before progressing to a fully powered RCT to assess effectiveness.   The secondary objectives are to assess trends in efficacy for whether the website:   1. improves child emotions and behaviours, their impact on the family, and parent mental health; and 2. increases use of services to support the child’s behavioural and/or emotional problems. |
| ***Outcomes and outcome measures*** | The primary outcome of acceptability and feasibility will be measured by both quantitative and qualitative measures. Quantitative measures include:   - Recruitment and retention of participants - Website usage - Survey of parents to determine whether key resources within the website were used by parents   Qualitative measures include:   - Semi-structured phone interview to assess parent experience using the website, including any problems associated with the website.   Secondary outcomes will be measured by:   - Child behaviour via parent reported measure using the strengths and difficulties (SDQ) and impact supplement - Parent mental health using the Depression, Anxiety and Stress Scale (DASS-21). - Health service use for their child’s behavioural and/or emotional problem. |
| ***trial Population*** | Up to 60 parents of two-12 year old children with behavioural and/or emotional problems, who have been referred to a paediatrician for management of this problem. The Website group and control group will have 30 participants in each group. |
| ***description of sites enrolling participants*** | Three private paediatric clinics in Geelong   - Geelong Paediatric Group - Geelong Children’s Clinic - Barwon Paediatrics   These sites are the only three private paediatric clinics in Geelong, in a regional centre with no generally accessible public paediatric clinics. |
| ***description of Interventions*** | The intervention consists of two components:   - FindWays co-designed website. FindWays is a new website designed to help parents find ways to help their child’s behaviour and emotions before seeing a paediatrician. It lists strategies the parents can try at home, online resources, and local providers who can help with a child’s behaviour or emotions. It was designed with parents living in Geelong (HREC 2019.160) - Prompts. Parents in the website Group will receive timed prompts to remind them about the website, provide a link to the website, and briefly describe the website.   The control group will receive standard care. This means they will not have access to the website but will still be able to access their usual supports. |
| ***TRIAL Duration*** | 12 months (August 2021 – December 2022) |
| ***participant Duration*** | 4 months |

# GLOSSARY OF ABBREVIATIONS

| ABBREVIATION | TERM |
| --- | --- |
| BEP | Behavioural or emotional problem |
| DASS-21 | Depression, Anxiety and Stress Scale |
| DHI | Digital health intervention |
| GCC | Geelong Children’s Clinic |
| GPG | Geelong Paediatric Group |
| RCT | Randomised Controlled Trial |
| SDQ | Strengths and Difficulties Questionnaire |
| SEIFA | Socio-Economic Indexes for Areas |

# INVESTIGATOR AGREEMENT

I have read the protocol entitled “FindWays: testing a co-designed website for parents to find ways to help their child’s behaviour or emotions.”.

By signing this protocol, I agree to conduct the clinical trial, after approval by a Human Research Ethics Committee or Institutional Review Board (as appropriate), in accordance with the protocol, the principles of the Declaration of Helsinki and the good clinical practice guidelines adopted by the TGA [Integrated Addendum to ICH E6 (R1): Guideline for Good Clinical Practice E6 (R2), dated 9 November 2016 annotated with TGA comments].

Changes to the protocol will only be implemented after written approval is received from the Human Research Ethics Committee or Institutional Review Board (as appropriate), with the exception of medical emergencies.

I will ensure that trial staff fully understand and follow the protocol and evidence of their training is documented on the trial training log.

| **Name** | **Role** | **Signature and date** |
| --- | --- | --- |
| Daniel Peyton | Chief Investigator | [signed D.P.] 08/06/21 |

# ADMINISTRATIVE INFORMATION

# Trial registration

# Trial registry

The trial will be registered with the international clinical trials registry platform before the first participant is recruited.

# Sponsor

| **Trial Sponsor** | **MCRI** |
| --- | --- |
| **Contact name** | Daniel Peyton |
| **Address** | **RCH, 50 Flemington Road, Parkville, 3052** |
| **Sponsor-Investigator** *(where applicable)* | **Yes** |

On behalf of the Sponsor, MCRI, the Sponsor-Investigator leading the trial will undertake and/or oversee those Sponsor responsibilities delegated by the Sponsor. The delegated Sponsor responsibilities are documented in Appendix 1 Division of Sponsor responsibilities.

# Expected duration of study

The study is expected to begin recruitment 02/08/21. Recruitment is expected to last for 3 months. Baseline measures will be assessed with a follow up survey at 4 months post randomisation. Final outcome data is projected to be collected by 31/3/21

# Contributorship

| **Name** | **Summary of contribution** |
| --- | --- |
| **Dr Daniel Peyton** | **Chief Investigator** |
| **Professor Harriet Hiscock** | **Supervisor** |
| **Dr Naomi Hackworth** | **Co-supervisor** |
| **Dr Greg Wadley** | **Co-supervisor** |

Dr Daniel Peyton is the chief investigator and is a general paediatrician. This study forms part of his PhD. He will be in charge of coordinating the day-to-day running of project activities. He will also be in charge of collecting data, analysis of data, liaising with research sites, consenting families and publication of the results.

Professor Harriet Hiscock (paediatrician, NHMRC Practitioner Fellow) is group leader, Health Services at MCRI and Director of the Health Services Research Unit at RCH. She is Dr Peyton’s primary supervisor and brings 20 years of experience in the design, implementation and evaluation of interventions for child health problems. She has supervised 7 PhD students to completion. She has co-authored past papers on intervention trials and pilots.

Dr Greg Wadley is a Senior Lecturer in the School of Computing and Information Systems at The University of Melbourne. He has studied the design and use of technology for health, psychological wellbeing and social interaction. Dr Wadley is a co-supervisor, and will assist with analysis and publication of the results. Dr Wadley has been involved with the intervention co-design and development.

Dr Naomi Hackworth is a psychologist, Acting Executive Director, Raising Children Network and Honorary Senior Research Fellow, LaTrobe University. She has over 20 years’ experience in child and family health and development and family functioning. She is expert in health communication, knowledge translation, research, evaluation and the successful development and uptake of digital platforms (websites, apps, webinars) in support of positive parenting. She leads content development for the Australian-government funded parenting website the Raising Children Network (raisingchildren.net.au) which produces and disseminate evidence-based resources to mass parent audiences.

# Stakeholder involvement

This trial has engaged with stakeholders in three ways. Firstly, paediatric clinics, who will form the primary location for this intervention in the future, have been involved in the development of this protocol, particularly around screening and recruitment. Both administration staff and the clinic paediatricians were involved in this process. Their involvement began in 2019 with design of the qualitative study “Understanding health service use, and interim-service preferences, for children with behavioural or emotional problem referred to see a paediatrician: A qualitative study” (HREC 2019.160). Secondly, allied health providers (psychologists, occupational therapists, parenting program providers) have been consulted in the design of the platform and content within the intervention. Again, this involvement began in 2019 as part of CI Peyton’s PhD. Finally and most importantly, parents have contributed to the design and development of the intervention. The parents’ influences span three co-design sessions, of up to four parents in each session, and 16 qualitative interviews with parents waiting to see a paediatrician in Geelong for their child’s behaviour or emotions. Their needs and preferences guided the content of the website, the user interface of the website, and the website functionality.

# INTRODUCTION AND BACKGROUND

# Trial rationale and aim

The aim of this study is to assess the acceptability and feasibility of a new co-designed website. This website, called *FindWays*, is designed to help parents of children aged 2-12 years with a behavioural or emotional problem, find evidenced-based strategies or local supports to help their child, while waiting to see a paediatrician. We will also assess trends in efficacy for outcomes including child behaviour and emotions, parent mental health, family impact and health service use and costs.

# Background

Behavioural and emotional problems

Behavioural and emotional problems (BEPs), such as disruptive behaviour, anxiety and mood problems, affect one in three Australian children.^1^ These problems can have both short and long term adverse consequences, and if left untreated, may worsen over time. Short term consequences include difficulty with child peer relationships, reduced educational achievement, and school suspension whilst for parents the consequences include poorer mental health and inter-parental conflict. Long term, child mental health disorders are the strongest predictor of adult mental health disorders.^2^ Their costs to the education, health and justice systems are high.^3^

Fortunately, there are evidence-based treatments available that have been shown to improve child behavioural and emotional problems, such as parenting programs (eg Tuning in to Kids) and online parenting interventions (eg computer assisted cognitive behaviour therapy – BRAVE online). These interventions can be delivered through individual therapy face to face, in group sessions, or self-directed through an online program. Often, they target the intervention towards the parent. As the parent is the largest modifiable factor in a child’s behaviour. Despite these evidence-based interventions for behavioural and emotional problems, very few parents of children with a mental health disorder will use them. Instead, parents often turn to the paediatrician for mental health care for their child. The paediatrician is the largest provider of longitudinal care of children with a mental health disorder. This is despite the fact that generally paediatricians do not deliver evidence-based behaviour management therapy or are trained in cognitive behavioural therapy. Instead, they will diagnose disorders, prescribe medication, re-refer to other providers, and exclude rare medical conditions.^1^ With few publicly funded paediatric services available, there are long waitlists in excess of 6-12 months for BEPs. Private paediatric services may see children sooner but are expensive and difficult to access for rural families. However, long waiting times preclude timely access to such support, especially for families who cannot afford private care. Anecdotally in Geelong, and in many other locations, many private paediatricians have closed their books to new referrals for behavioural and emotional problems.

What parents want while waiting to see a mental health specialist

Parents waiting to access mental health services want information while they wait. This might be directed towards a better understanding how the mental health system works, or access to treatment to help their child while they wait, such as group therapy, an online intervention or telephone support.^4^

From my qualitative interviews with parents in Geelong, inductive content analysis revealed parents want to understand whether their child’s problem was normal or not, what they could try while they waited to see the paediatrician, what services could help their child’s problem, and a list of available expert help in their community.

Why a digital health intervention might help

Digital health interventions (DHIs) pose a potential solution to address these gaps raised by families. DHIs are desirable for the following reasons: they facilitate the rapid exchange of tailored information to consumers, are scalable, parents like them, they are accessible on demand, and generally thought to be cost-effective.^5^

Digital health interventions have been shown to improve mental health knowledge among parents of 2-12-year-olds with BEPs but they have never been shown to improve uptake of mental health services.^6,7^ This is of concern because improving parent mental health knowledge without improving uptake of evidence-based services for a child, is unlikely to change a child’s behaviour.

Design and evaluation of a Digital health intervention

There is no gold standard of digital health intervention design or evaluation. However, there are a number of existing frameworks and recommendations that promote an iterative approach to design and evaluation.^8,9^ Michie et al. ^10^ describe a person-centred, iterative approach with mixed methods feedback that refines the DHI to the needs of the user, through every phase of development. This includes from initial usability testing, to feasibility, through to efficacy, effectiveness, economic evaluation and implementation research with continuous, real-time monitoring of the DHI’s expected outputs.^10^ This process is well described within a systematic review of evaluation methods for DHIs.^11^ Overall, they describe a model for evaluation occurring in multiple phases: i) design phase, ii) pretesting phase, iii) pilot study, iv) pragmatic trial phase, v) evaluation phase, and vi) postintervention phase.^11^

The components of a pilot feasibility trial vary but there is a defined need to assess the acceptability including usability, and the feasibility of the implementation including whether the DHI works as intended.^12^ The WHO describes this testing phase of the prototype as a way of understand whether the DHI “works as intended in a given context” and whether it “can be used as intended by users”.^12^ A mixed methods evaluation is best to evaluate these outcomes.

The role of prompts.

One challenge that plagues DHIs is uptake and engagement. A DHI may not be used by research participants, or when used once, rarely used again. There are well described studies that document the reasons, which can include lack of awareness, motivation, suitability, and perceived usefulness of the DHI. We know that regular prompts can improve uptake, and address some of these identified barriers. However, the ideal frequency, timing and message content is unknown. As such, these aspects of prompts will be assessed within the remaining co-design sessions of the project (HREC 2019.160).

Hypothesis.

We hypothesise that FindWays is an acceptable and feasible way of potentially helping parents find ways to help their child’s behaviour and emotions while they wait to see the paediatrician. As this is a pilot RCT, it will not be powered to assess effectiveness of parent, child or health service use outcomes.

**Significance**

Following the pilot RCT and PhD, I plan to conduct a fully powered, community-based RCT to determine the effectiveness and cost-effectiveness of this intervention in reducing the need for paediatrician services and improving child and parent outcomes.

In the long term, the FindWays website is potentially significant because: i) it may help families improve their child behaviour and emotions at home, or ii) find and engage with available evidence-based services earlier.

If this DHI can link some families to the right treatments, without seeing a paediatrician, it will increase the efficiency of the health system. With fewer families on waitlists, children with more complex or severe problems may be seen sooner. The overall capacity of the health system may be increased as we expect families to access group parenting programs, and scalable online programs, where there is greater capacity to see more children than through individual face to face appointments with a specialist.

**Long term outcomes**

Knowledge translation

Outcomes of the future fully powered RCT, if effective, could lead to transfer of this platform and model of care to other paediatric hospitals’ waitlists for child BEPs, through open dissemination of the platform and development of a simple flowchart assisting clinicians who triage referrals. This process would be facilitated by promoting the intervention through state and national organisations representing paediatricians (eg Victorian Paediatric Clinical Network, Neurodevelopmental and Behavioural Paediatric Society of Australasia, the Royal Australasian College of Physicians). In the future, I plan to partner with existing online consumer health information providers (eg Raising Children Network – co-located at MCRI) to host and promote the website.

# Risk/Benefit assessment

# Known potential risks

There are several short and long term risks that need to be considered for a trial of a digital health intervention attempting to help parents find strategies and services to help their child.

**Quality of information and services**

The first short term risk relates to the content contained within the website. This includes whether the parenting strategies are accurate, and whether the services are available and right for the families. To reduce this risk, the parenting strategies reference existing information sources online, such as the Raising Children Network, Beyond Blue website, the Diagnostic Standard Manual-V and UpToDate. All of the strategies and treatments within the website have been previously cited on one of the previous resources. All of the content has been written by Daniel Peyton, a general paediatrician. The content goes through a quality review process with review by another paediatrician and researcher at RCH. Video scripts are reviewed and approved by the paediatricians in Geelong. The content is also reviewed by Dr Hackworth (acting Director, Raising Children Network) to ensure the tone is appropriate for families. Parents of children with a behavioural or emotional problem will also review the content as part of the co-design process in June 2021.

The services within the website are all services available withing Geelong and advertised on publicly available websites. The psychologists are listed on Google, health services directory or are recommended by local paediatricians. The Occupational Therapists are listed as Medicare approved providers on the Find an OT website, having had additional accreditation to look after children with mental health problems. These providers are trained to diagnose and treat mental health conditions as part of their training. Parenting programs are run by the City of Greater Geelong. This is a regional service that specialises in parenting and parent education. Online interventions listed on the platform have RCT level evidence with 6 months outcomes supporting their use for behavioural or anxiety related problems. Any parent already has access to all of these services, without using the platform, and all of these services can be found online through a Google Search.

Parents will have a choice as to whether they want to pursue a parenting strategy or service while they wait to see the paediatrician. They are not being recommended to pursue it, the platform simply aims to facilitate access to relevant information, suitable to a child’s particular symptoms (as selected by the parent). The parents have a choice of strategies and providers based on the theory of patient centred care, which priorities patient preference.^13^

**Participant distress**

We do not anticipate causing major distress to parents. Some of the survey measures may be uncomfortable for parents to complete. To help reduce this risk we will provide a clear explanation of why the study is being done, why the information is collected, and provide warning to the parents about what questions they will be asked before recruiting the parent. We will also emphasise to parents that the information is confidential, voluntary and they are free to stop at anytime. The participants are also reminded, in the participant information sheet, that they can contact a member of the research team via email or through the research mobile numer . This phone number is a dedicated research phone that will be used to contact parents as required within the project. In an emergency, we have directed families to existing 24/7 support services for children’s mental health.

The interviews will be conducted by CI Peyton, who is experienced in clinical work with families and managing distress. There is also a minor risk of parents becoming distressed during the qualitative interviews, talking about their experiences using the website. If a parent does become distressed during an interview, it will be important to acknowledge that they are upset and give them an opportunity to talk about how they are feeling, and listen supportively and empathically. The family may prefer to take a break for a short period of time or to finish the interview on another day, or to withdraw from the study entirely.

If the family continues to be distressed, the researcher will offer further support, such as:

- Ask them what support they have at present, and discuss about other ways they might get support from family or friends
- Direct them to relevant support services, including (but not limited to): Lifeline (13 11 14), Beyond Blue (1300 22 46 36), and Kids Helpline (1800 551 800).
- If a family appears distressed but doesn’t want the researcher to assist in getting additional support, to the researcher will respect the families’ wishes.

This process was used successfully during CI Peyton’s qualitative interviews in early 2020 (HREC 2019.160)

**Long term risks**

One theoretical long-term risk exists from using the platform, which is whether parents feel they find the help they need from the website and linking them to alternative services, so they don’t proceed to see the paediatrician. It is worth noting that this attrition is common among paediatrician waitlists, and may represent the families finding alternative services or a resolution of the problems they were referred for. To reduce the risk of parents prematurely withdrawing from the waitlist, the website is framed around encouraging parents to always access the paediatrician, and the strategies and services provided within the website focus on giving parents strategies and services to try while they wait for an appointment with the paediatrician.

It is also worth noting a similar mental health service navigation website for teenagers found no adverse effects from their intervention in either their feasibility or efficacy trial.^14,15^

# Known potential benefits

The potential benefits of this website affect several of the stakeholders, specifically parents and children, administration staff and paediatricians. For each group, there are different potential benefits.

1. Providing parents with information they want while they wait. We know parents waiting for mental health treatment for their child want information on how the mental health system works and what they can do to help their child while they wait.^4^ We aim to provide this information to families, in conjunction with specific information to address parents needs and preferences, as based on the input of parents who have been interviewed in CI Peyton’s qualitative interviews. By making it easier to find relevant and useful information about services we may be able to reduce the stress and improve coping.^15^ Providing parents with additional information about the mental health system may also keep them better engaged with the system and prevent them from stopping their efforts to get help for their child.^16^
2. Families have frequently been rejected from paediatric services in Geelong due to high demand and little capacity of the paediatricians to meet this demand. Among parents who have been rejected, this intervention provides parents with viable alternatives while they continue to try and navigate the mental health system and find an available paediatrician with the help of their regular supports.
3. If the intervention does improve access to evidence-based professional support, then child behaviour may improve earlier and be less severe when they do see the paediatrician. This reduction in the severity of the child’s problem may reduce the need for pharmacological therapies when they see the paediatrician.
4. Anecdotally, the administration staff at clinics often provide advice to parents to try and help the family when the clinic cannot provide timely help. This website may offer a way to provide quality information to help families by providing information on local services, that does not rely on the subjective advice of the administration staff, who may not have any professional training in mental health support.

# Assessment of potential risks and benefits

Based on the above, it appears that the benefits of providing additional information about relevant strategies and services likely outweighs the associated risks. Many families, in previously published evidence and through my qualitative interviews with families (HREC 2019.160), have expressed preferences for additional information about strategies they can use to help their child and information about what interim services that may be suitable while they wait to see the paediatrician.^4^

# TRIAL OBJECTIVES AND OUTCOMES

# Objectives

# Primary objective

The primary objective of this study is to assess the acceptability and feasibility of: i) a co-designed website for parents of children aged 2-12 years old with a behavioural or emotional problems referred to a paediatrician for their first appointment; and ii) the study design and measures, before progressing to a fully powered RCT to assess effectiveness.

# Secondary objectives

The secondary objectives are to:

1. improves child emotions and behaviours, their impact on the family, and parent mental health; and
2. increases use of services to support the child’s behavioural and/or emotional problems.

**Table 1. Study Objectives, data sources, measures, data collection time points and outcomes**

| Primary and Secondary Objectives | Data Sources | Methods of collection | Data Collection Timepoints | Outcomes of Interest |
| --- | --- | --- | --- | --- |
| Acceptability – task completion | Parent Survey | online via REDCap | Baseline and 4 months | Whether parents accessed any of the resources within the website, and whether this changed the parent’s behaviour (eg help-seeking, implementing new strategies) |
| Acceptability - recruitment | Clinic database | Print copy of database | recorded at weekly intervals for the duration of recruitment | How many eligible patients were flagged by the paediatrician, how many were contacted by the clinic, and how many agreed to pass on their details to me and how many were randomised to the intervention/control. |
| Acceptability – retention and usage | Google Analytics | Manual data extraction from Google Analytics | Baseline and Weekly intervals for 4 months | Number of page views, individual sessions, time spent on each page.  Strategies viewed, provider profiles viewed, programs viewed. |
| Acceptability - recommendation | Parent Survey | online via REDCap | 4 months | Likert scale of NET recommender score from 0-10. |
| Acceptability – usability and safety | Qualitative Interview | Qualitative interview with parents over 20 minutes | 4 months | Understand the parent’s experience using the platform, barriers to use and any perceived adverse outcomes |
| Secondary outcome: Child Behaviour and emotions | Parent Survey | Control and website group surveys completed online via REDCap. | Baseline and 4 months | Strengths and Difficulties Questionnaire* (SDQ) measuring changes in behaviour and impact |
| Family impact of child’s behaviour and emotions | Parent survey | Control and website group surveys completed online via REDCap. | Baseline and 4 months | SDQ impact statement* measuring the impact of the child’s behaviour and emotions on their functioning and the family. |
| Secondary outcome: Parent mental health / distress | Parent Survey | Control and website group surveys completed online via REDCap. | Baseline and 4 months | Depression, Anxiety and Stress Scale* (DASS-21) measuring changes in parents’ symptoms |
| Secondary outcome: Demographics | Parent survey | Control and website group surveys completed online via REDCap. | Baseline | Collection of demographic statistics to look for trends in acceptability between demographics of interest. |
| Secondary outcome: Health service use | Parent Survey | Control and website group surveys completed online via REDCap. | 4 months | Survey question of services used in the past 4 months, number of times accessed and distance travelled, and out of pocket costs. |

* Validated measure

**Summary of the validated measures.**

Strengths and Difficulties Questionnaire

The SDQ^17^ is a brief psychological assessment tool developed for 2-17 year old children and adolescents which consists of 25 items on psychological attributes. The SDQ has been well validated^18^ and is consistent over time in an Australian context^19^.

Strengths and difficulties Questionnaires with impact statement

This extended version of the SDQ asks additional information about the duration, level of distress, level of impairment, and the degree of burden their behaviour or emotions has on others, including their family.^20^

Depression, Anxiety and Stress Scale

The DASS-21 is a 21 question survey for adults, that measures emotions of depression, anxiety and stress. The DASS-21 has also been validated and there are accompanying norms for the Australian population.^21,22^a

# TRIAL DESIGN

# Overall design

A pilot randomised controlled trial will be implemented to assess the acceptability and feasibility of the FindWays website and compared trends in child and parent related outcomes, compared to a standard care control group,

*Design:* Pilot RCT comparing the FindWay website intervention group with a control group.

*Intervention*: FindWays website is a co-designed online service navigation platform and website providing evidence-based parenting strategies. Informed by qualitative study (HREC 2019.160) and co-design workshops, the website provides content on children’s behavioural and emotional problems. Specifically, this content includes strategies to help specific behaviour or emotional problems, describes providers and programs that can help child behavioural or emotional problems, and a list of local community services and individual professionals who can see children with a mental health care plan. Parents will have access to the intervention for 4 months.

*Control*: A standard care control group was chosen to measure the realities of trying to access paediatric help for the child’s behavioural or emotional problem. This also allows us to compare trends in efficacy of secondary outcomes between the two groups.

*Sample size:* There are no published data on the optimal size of a pilot RCT to assess acceptability and feasibility. However, prior studies conducted by this team have included between 15-30 participants in each trial arm, and the Melbourne Children’s Trial Centre has suggested a minimum of 20 participants. As such, I will aim to recruit 60 parents, with 30 in each arm.

*Setting:*Three specialist general paediatric outpatient clinics in Geelong, Victoria. There are no public paediatric outpatient clinics available to Geelong children, unless the family identifies as ATSI or migrant background. There are three private clinics available in Geelong, of which bulk billing is paediatrician dependent on a case-by-case basis.

# Trial population

The trial population will be all eligible parents of children, aged two-12 years. with a behavioural and/or emotional problem, who are referred to a paediatric clinic for their first review. These paediatric clinics will be either Geelong Children’s Clinic, Geelong Paediatric Group or Barwon Paediatrics. The referred child can either be accepted or declined by the clinic.

We expect to recruit at least 20 parents every month for three months. This estimate is based on the high number of monthly referrals for behavioural or emotional problems in 2-12 year old to these clinics (estimated to be 20 referrals every week across the 3 clinics). Further, the number of accepted referrals who were recruited within CI Peyton’s qualitative study (HREC 2019.160), was 5 parents per week at each study site.

No power calculation has been done as the trial is not intended to assess the effectiveness of the intervention. Typically, acceptability and feasibility trials recruit around 20 participants, though 60 will give us a stronger indication of difference in acceptability between the intervention groups, and a larger number of eligible participants for the qualitative component of the study, which ideally requires around 20 participants to reach data saturation.

Exclusion criteria are listed in the table below. These criteria are justified because: i) children with a diagnosed intellectual disability or autism spectrum disorder require specialised treatments that are not listed within the platform; ii) children in out of home care are especially vulnerable and again require specialised programs with an emphasis on attachment and trauma; iii) any child requiring an urgent review (eg high severity or possibility of medical cause) is less likely to gain benefit from the website due to the shorter time frame waiting for a paediatrician review, and may be less likely to benefit from the programs and interventions listed in the website because of the increased severity.

Parents are the sole focus of the intervention, and the only source of outcome data. Children are not directed to use the website or participate in the collection of any measures. Only one parent will be consented for each child referred to the clinic.

**Table 2**. Inclusion and exclusion criteria

| *Inclusion criteria* | *Exclusion criteria* |
| --- | --- |
| Parent of child aged 2-12yo  BEP listed as primary concern on the GP referral letter for first appointment, i.e.:   - anxiety (eg excessive stress, worry), - behavioural problems (eg disruptive, defiant, hyperactive) - mood problems (eg depressed, emotional dysregulation) | Child in out of home care  Parent reported child intellectual disability or previously diagnosed ASD  Paediatrician defined need for an early review (eg to exclude medical problems) |

# Eligibility criteria

Participants will be assigned to a randomised trial intervention only if they meet all of the inclusion criteria and none of the exclusion criteria.

# Inclusion criteria

Each participant must meet all of the following criteria to be enrolled in this trial:

- Is a parent or carer of a child aged 2-12 years at the time of randomisation
- Has been referred for a first appointment with a paediatrician to manage a behavioural or emotional problem
- Has a behavioural or emotional problem as listed for review on the referral letter
- Provide a verbal consent that is signed and dated by the researcher.

# Exclusion criteria

Patients meeting any of the following criteria will be excluded from the trial:

- Has a parent reported diagnosis of an intellectual disability or Autism spectrum disorder
- Child in out of home care
- The paediatrician decides the child needs an early review (such as for a severe problem or to exclude a medical problem).

# Screen failures

Screen failures are defined as participants who consent to participate in the trial but who are found, during the screening procedures, to be ineligible to continue in the trial. They therefore do not receive the intervention / are not randomised.

# Recruitment and identification of potential participants


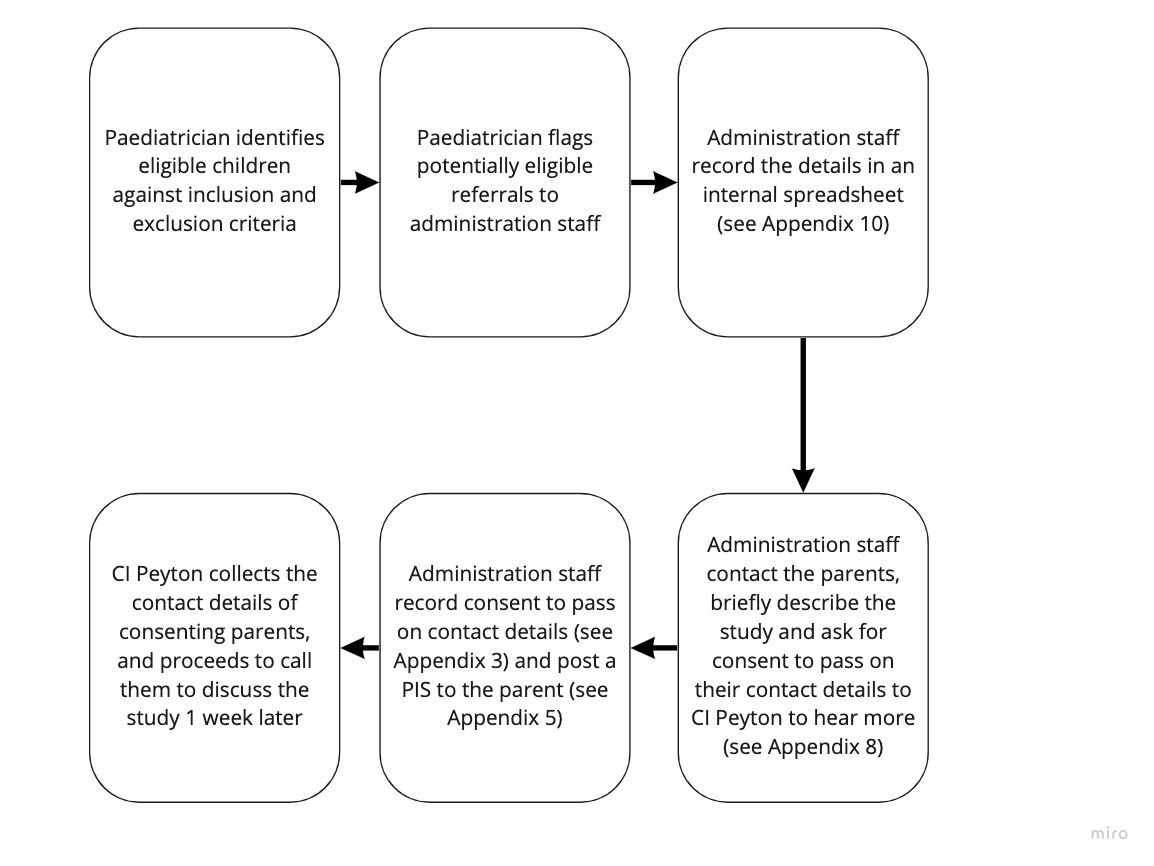


Figure 1. Summary of recruitment process.

**Screening eligible referrals**

Children referred for a primary problem of behavioural and/or emotional problems will be evaluated by the paediatrician, based on the information in the GP referral, against the above inclusion and exclusion criteria. Children with two or more problems will still be eligible, as long as 1) the primary concern is a behavioural and/or emotional problem, 2) they do not require an urgent review as per the paediatrician’s regular care triage assessment, and 3) there is no indication from the referral they meet exclusion criteria.

**Recording recruitment rate**

Once the paediatrician has flagged a child as potentially being eligible, the clinic administration staff will record the potential eligibility on an internal database to keep track of who has been screened and when they have been called. Administration staff will then telephone the family to request consent to pass on the parent’s contact details to CI Peyton.

**Phone parents direct**

Administration staff at the clinics will call the family to let them know their referral has been received, and whether they have been accepted onto the waitlist, and if so what their estimated appointment date/time. At the end of this conversation, the administration staff will initiate the telephone script. For those parents who wish to hear more about the study, the administration staff will take and record verbal consent from the parent to pass on the parent contact details (parent name, email address, phone number and postcode) to CI Peyton.

Those who do not consent to passing on their contact details will have their non-consent recorded without any identifying information, to help keep track of numbers of those who do consent and those who don’t consent to pass on their personal information.

**Clinic to post parents a PIS**

For those parents who do consent to pass on their contact details to CI Peyton, their contact details will be recorded, along with a record of their verbal consent. At this time, the clinic will also post the parents a copy of the participant information statement.

**Passing on personal details to CI Peyton**

This record of verbal consent and contact information will be handed to CI Peyton by administration staff in hard copy on a weekly basis. This will not contain any information regarding the title or nature of the study. This list will be collected in person by CI Peyton every week from all three clinics. This list will be transported to MCRI and stored in a locked filing cabinet. Only CI Peyton will have access to this information. CI Peyton will then assign a unique ID to each family, and contact the families as described in section 6.5.

One week after the parents have consented to passing on their contact information, CI Peyton will contact the parents. One week will allow adequate time for the parents to receive the participant information statement in the mail. Australia Post has an estimated delivery time of 3-4 business days across the whole of the city of Greater Geelong.

When contacting the parents, CI Peyton will text the participant to let them know when he will call them. This will give an opportunity to change the contact time to suit their needs. During the phone call, CI Peyton will tell them more about the study, check they received the PIS and answer any questions.

If CI Peyton fails to contact a primary caregiver after 3 attempts, he will send them an email asking them to contact the study team, or their clinic to let them know about their preference. CI Peyton will not phone, or email, the parent again unless they request further contact with the research team through the above avenues.

# Consent

One week after the parent provides their clinic with verbal consent to share their contact information, CI Peyton will phone the family, check they have received the participant information statement, briefly describe the study and ask them if they have any questions. CI Peyton will then check inclusion and exclusion criteria and obtain verbal consent from the participant.

CI Peyton will conduct the informed consent discussion and will check that the parent comprehends the information provided. CI Peyton will answer any questions about the trial.

The parent will be invited to provide verbal consent. Consent will be voluntary and free from coercion. CI Peyton who conducted the consent discussion will sign the informed consent form.

The participant will then be sent the baseline survey, via SMS or email according to parent preference. Once the baseline survey on REDCap has been completed, they will be randomised to a trial arm.

Those parents who do not consent will be asked if they are willing to tell the researcher their child’s age in years and postcode. This will help determine if there are specific child ages or postcodes (which we will use as a proxy for their SEIFA scores) are rejecting the trial. The number of parents not consenting will be recorded to as a measure of the recruitment rate.

# INTERVENTION

# Intervention arms and the intervention

This trial has two arms: i) an intervention arm and ii) control arm consisting of standard care.

The intervention consists of access to a new, co-designed website. This website, FindWays, offers parents relevant, credible and specific information on behavioural and emotional problems. This will hopefully help the parent find additional ways to help their child while they wait to see a paediatrician, over and above their usual supports (eg GP). This information includes different parenting strategies parents can try at home, learn what different professionals or programs can do to help, and where to find available professional help.

This website contains 60 pages of content, hosted on WebFlow. Each parent will be provided a unique URL to access the website. The website does not store any identifying information about the child. The website does not record any identifying information about the parent. The parent can select specific ages and problems that are of interest, similar to any other mental health website, to find tailored information relevant to their child.

The website was designed, based on existing theory for development of digital health interventions, and on evidence generated by my own research and from previously published research.

The theory for design, development and evaluation of a digital health intervention is as described by the World Health organisation, and the centre for eHealth research and disease management.^8,12^ This describes an iterative approach, of contextual enquiry, understanding user needs and functionality within the DHI, design and prototyping, progressing to evaluation through pilot trials, and eventually to larger RCTs to test real world effectiveness.^8^ The intervention was also influenced by existing evidence on the benefits of incorporating participatory design theory and behaviour change theory into the intervention.^23–26^

Using participatory design methods, the intervention was co-designed with parents. These parents were recruited from Geelong, as part of a pre-existing study on understanding parents’ needs and preferences when they were referred to a paediatrician in Geelong (HREC 2019.160). The design and content of the FindWays website was influenced by the findings from the inductive content analysis of the interviews and three co-design workshops.

The control group will receive standard care. This care is provided by usual providers (eg GP, existing online resources, teachers at school).

# Intervention(s)

# Measurement of participant compliance

Compliance with the intervention will be measured through the parents’ usage of the website.

Participants will be given a unique URL to access the FindWays website. Their unique URL will be securely stored alongside their participant ID, in the REDCap database. The only way to link the individual website URL (and their usage data) and the participant is through the REDCap database.

Google analytics data will be recorded for each unique URL. Usage data, as is routinely collected by Google analytics, will include:

- Pages visited
- Time on each page
- Number of sessions
- Number of days visiting the website
- External links clicks
- Device (eg phone, tablet, desktop)

This google analytics data will then be manually entered into REDCap database by CI Peyton.

No identifying information is stored on the website, as there are no log ins, child information, user accounts of profiles.

# Discontinuation from trial intervention

The intervention group will have ongoing access the FindWays website. This will allow potential opportunity for longer term measurement of outcomes related to the acceptability and feasibility, and trends in efficacy.

If the research team decides to measure longer term outcomes (eg at 12 and 24 months) the researchers will submit an amendment to the HREC.

# RANDOMISATION AND BLINDING

# Randomisation and concealment mechanism

A statistician not directly involved in the analysis of the trial results will prepare the randomisation schedule. The randomisation schedule will be created by computer-generated random numbers, before the first participant has been recruited. The participant cohort will be stratified by child age (2-6yo and 7-12yo) and clinic (eg GPG, GCC and Barwon Paediatrics). Within each strata, permuted block randomisation will be used to ensure balance between the Website and control group. A randomly generated sequence of block sizes containing 2, 4, or 6 participants will be used. This will help ensure balance in numbers between Website and control groups, prevent potential confounders of age or clinic type impacting the measurement of outcomes, and prevent any predictability in Website allocation.^27^ The schedule will be held by an independent statistician, and allocation will not be revealed prematurely to CI Peyton. Because of these procedures, the research team will be unable to predict which group the participant will be allocated to.

Both CI Peyton and the participant will become aware of which trial arm they were allocated to after randomisation. The participant and researcher will not be blind to their intervention status because it is impossible to blind a novel website intervention to participants. The administration staff and paediatricians will not be notified by the researchers of the intervention status of the participants.

# Breaking of the trial blind

# On trial

Participants and the research team will be unblinded to the intervention. Their allocation will be revealed after randomisation, when they are referred to access the platform.

# TRIAL VISITS AND PROCEDURES

# Trial timeline


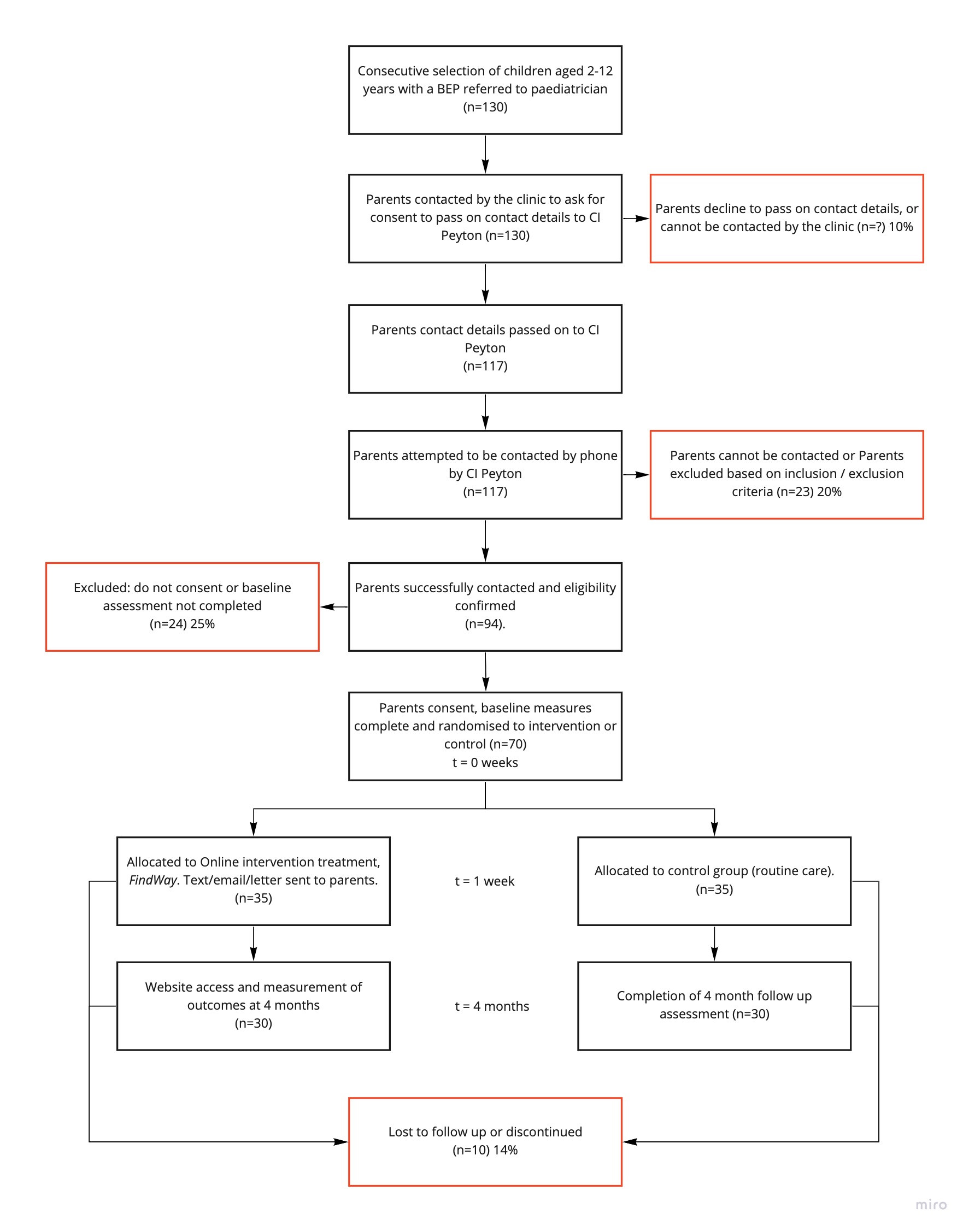


Table 2. Research Project Timeline

|  | 2021 | | | | | 2022 | | | | |
| --- | --- | --- | --- | --- | --- | --- | --- | --- | --- | --- |
|  | Aug | Sep | Oct | Nov | Dec | Jan | Feb | Mar | Apr | May |
| Participants 1-20 | Baseline data | Intervention | | | 4 month outcomes measured |  |  |  | Analysis and write up | |
| Participants 21-40 |  | Baseline data | Intervention | | | 4 month outcomes measured |  |  | Analysis and write up | |
| Participants 41-60 |  |  | Baseline data | Intervention | | | 4 month outcomes measured |  | Analysis and write up | |

# Schedule of assessments

Table 3. Sequence of enrolment, consent and measures

|  | **TRIAL PERIOD** | | | |
| --- | --- | --- | --- | --- |
|  | **Enrolment** | **Allocation to intervention** | **Post allocation** | **Close-out** |
| **TIME POINT**** | ***-t_1_*** | **0** | ***t_1_*** | ***t_x_*** |
| **ENROLMENT:** |  |  |  |  |
| **Eligibility screen** | X |  |  |  |
| **Informed consent** | X |  |  |  |
| **Allocation to intervention** |  | X |  |  |
| **INTERVENTIONS:** |  |  |  |  |
| ***Website group*** |  |  |  |  |
| ***Control group*** |  |  |  |  |
| **ASSESSMENTS:** |  |  |  |  |
| ***Child behaviour, parent mental health survey*** | X |  |  | X |
| ***Demographics survey*** | X |  |  |  |
| ***DHI usage as measured by Google Analytics*** |  |  | X  (continuous) | X |
| ***Acceptability survey (FindWays group only)*** |  |  |  | X |
| ***Qualitative interview (Optional consent from FindWays group only)*** |  |  |  | X  (optional) |
| ***Health service use survey*** | X |  |  | X |

# Description of procedures

Once parents have been contacted by CI Peyton and consented to taking part in the research, the parent will then be sent a SMS with a link to a REDCap survey.

This REDCap survey will be filled out by the parent, where they will record their baseline demographics, and validated measures of child behaviour, parent mental health, family impact and health service use.

When this survey has been completed, they will then be randomised to the intervention or control group. Once they have been randomised, the parents will be notified by SMS of their treatment group allocation.

The participants in the intervention group will then be sent a text message containing their unique link to the FindWays website. Those in the Website group will then be free to access the intervention at their leisure.

On a semi-regular basis, parents in the intervention group will be sent a reminder to access the website. These reminders also contained some brief information about the purpose of the website.

Table 4. Schedule of SMS reminder prompts to parents

|  | **Trial Period** | | | | | |
| --- | --- | --- | --- | --- | --- | --- |
|  | Allocation to intervention | Post allocation | | | | |
| Time point | 0 | 1 week | 2 weeks | 4 weeks | 2 months | 3 months |
| SMS reminder |  | X |  | X |  | X |
| Email reminder |  |  | X |  | X |  |

Parents can request at any time to stop the prompts, either by email or text message. This is stated to parents in the PIS.

At the end of the 4-month trial period, parents will be texted and/or emailed a link to the final survey. They will receive 3 reminders to complete this survey, then CI Peyton will call the parent and offer to complete the survey over the phone. If this is unsuccessful, we will stop and consider the parent as having dropped out.

For parents in the Website group, they will be contacted via SMS to participate in a telephone semi-structured interview. CI Peyton will conduct all the phone interviews as per the interview guide. The interview will last approximately 30 minutes. Dr Peyton has conducted qualitative interviews before (HREC 2019.160), attended qualitative methods training through University of Melbourne courses with Prof Lynn Gillam and attending workshops as part of his PhD Mental Health Program. This interview is designed to better understand the parent’s experience using the platform, including understanding any adverse events (including rare safety issues) that occurred because of using the FindWays website.

We will request permission to audio record the interviews from caregivers. The interview will be recorded on Dr Peyton’s digital dictaphone whilst conducting the interview on speaker phone in a walled office at MCRI or at Dr Peyton’s home office. If the respondent does not consent to an audio recording of the interview, CI Peyton will take notes during the interview, and use these written notes for data analysis. All audio recordings will be transcribed verbatim by either CI Peyton or Outscribe. Outscribe is an on-demand transcription Australian based service, used previously by researchers at MCRI. The information in the interview will be related to the use of the platform, and not asking about sensitive health information.

Audio recordings will be retained after transcription for 5 years. They will be stored on a MCRI network restricted access folder on a password protected computer. Audio recordings are required to be stored as the source data of the qualitative interviews to ensure an audit trail is maintained.

# Withdrawal of consent - participant withdraws from all trial participation

Participants are free to withdraw from the trial at any time upon request. If known, a brief reason will be recorded on the participant database. All parents in the Website group who withdraw will be asked if they are still willing to participate in the 20-minute final interview to understand their experience using the platform. Withdrawing from the trial will not affect their access to standard treatment or their relationship their paediatrician. If they withdraw, they will no longer have access to the FindWays website.

# Losses to follow-up

A participant will be considered lost to follow-up if they fail to return 4 month follow up measures and are unable to be contacted by trial staff. Before a participant is deemed lost to follow-up, CI Peyton will attempt to contact the family 3 times via SMS and then once by phone. These contact attempts will be documented in the participants REDCap database.

Should the participant continue to be unreachable, he or she will be considered to have withdrawn from the trial with a primary reason of lost to follow-up.

# Replacements

Participants who sign the informed consent form and are not randomised / assigned trial intervention may be replaced. Participants who have been randomised / assigned trial intervention may NOT be replaced.

# Trial Closure

A participant is considered to have completed the trial if he or she has completed all phases of the trial including completing the final survey as detailed in the Schedule of Assessments.

# Continuation of therapy

We will not restrict participants’ access to the FindWays website after the trial has ended. Participation and non-participation in the website will not affect their progression through the wait list to see the paediatrician.

# SAFETY MONITORING AND REPORTING

This website-based intervention does not meet the criteria for an investigational medical device, according to the definition of the [Therapeutic Goods Administration](https://www.legislation.gov.au/Details/C2019C00066). However, it is still important to consider the ethical implications of the website’s use. Specifically, this relates to the potential for the website to impact participant safety, participant distress, and concerns raised about the safety or welfare of the child.

**Participant safety**

This intervention and trial could impact patient safety through two potential hazards: i) low risk of hazard from the intervention, and ii) risks associated with the privacy of the information collected.

**Low risk of hazard from the intervention**

The potential for hazard from the intervention is low, as the is unlikely to be any safety issue beyond a trivial or minor problem. This is because the intervention does not provide diagnostic advice or seek to rate the degree of their child’s problem or disorder, nor does it provide novel treatment methods. Within the intervention, the parents self-select a behavioural or emotional problem that is most relevant to their child (similar to what occurs on freely available websites like Beyond Blue and Raising Children Network), and the information provided within the intervention is based on existing, publicly accessible strategies referenced from reputable sources online.

**Risks associated with privacy**

The second issue potentially affecting patient safety relates to the storage of confidential information and the parent’s privacy. Participant Information Statements indicate that all information provided to the research team is confidential and will not be made available to any other agency. Any information collected for this research project that can identify participants will be treated as confidential. The information will be stored on a secure REDCap database. Handwritten information sheets (eg record of verbal consent) will be stored separately in a locked filing cabinet at MCRI. These information sheets will not be linked to the participant health data. Participant data will be recorded using a unique ID. This unique ID will be stored on a secure password protected server at MCRI that links the participant names with their unique ID. These information sheets will not be linked to the participant data.

**Participant distress**

Although we expect participants will not be at major risk of becoming distressed during this research, the participants could become distressed during this trial through three potential ways: i) filling out surveys on their child’s behaviour or their own mental health symptoms, ii) inconvenience and associated frustration from the potential provision of inaccurate information on the website, and iii) within the qualitative interviews at the end of the study.

Distress filling out surveys

Completion of the survey may raise issues which are uncomfortable (confidence and competence in care), upsetting, or frustrating for participants. To reduce the potential for distress to families, the researcher will provide clear explanations about why the research is being conducted, how the information will be used, and the kinds of questions that will be asked in the questionnaire prior to recruiting the family. They will also emphasize that:

- All information families provide is confidential (unless we are legally compelled to disclose)
- Families do not have to answer any questions they don’t want to
- It is OK to stop the survey at any point.

Potential for inconvenience or frustration using the intervention.

When using the intervention, some families may trial strategies that are ineffective, or try to access providers who are unavailable. This is an unfortunate aspect of the current mental health system, where it is impossible to be certain of what will help or who is available. To help reduce the risk of any failed strategies or services, all the strategies are evidence-informed, sourced from reputable sources and fully referenced. And, all the providers availability data has been sourced directly by Dr Peyton, and will be updated halfway through the recruitment period, by contacting the providers direct. Participants will have the option to go back and talk to their GP for additional support, if they become frustrated by a lack of success using the intervention. The participants will also have the option to withdraw from the trial at any time, without reason and it will not affect the care they receive from the paediatrician.

Qualitative interviews specific risks

Four months after starting the intervention, the parents in the Website group who gave optional consent to participate in a semi-structured interview of 30 minutes, will be contacted (see interview guide). Completion of this qualitative survey poses 3 possible ethical complications: (i) the parent becomes distressed during the phone interview, (ii) parent’s description of them using the FindWays website indicates need for urgent intervention (eg perhaps because the child’s situation has deteriorated over the past 4 months); or (iii) parent may say something about using the website that raises concerns about child’s safety and welfare (eg a protective concern).

While unlikely, it’s possible that participant distress may arise as parent’s talk about their experience using the platform. There is a small possibility that this may be uncomfortable, upsetting and frustrating. In this research setting, there are additional methods available to prevent and manage the potential for parental distress. Specifically, the parents can stop the interview at any time, they can avoid answering any questions they don’t want to, and that it is OK if they choose to do so.

If any parents become distressed during the phone interview, we will adhere to the following initial procedure:

1. acknowledge they are upset
2. talk to them empathetically and listen to their concerns
3. give them the opportunity for a break or withdraw from the study

If this is unsuccessful, and the participant becomes more distressed, we will escalate the procedure to:

1. Terminate using the interview guide;
2. CI Peyton will attempt to calm the participant and direct them towards appropriate, available resources, depending on their cause of distress; These resources include
   1. Asking about support persons specific to that person (eg family, friends) who they can discuss their concerns with.
   2. The GP who made their referral
   3. Mental health resources – national help lines (Parentline 132289) or online self-help resources (Raising children network, Beyond Blue)
   4. Ask if we may call them in a couple of days to check in on how they are going, where Investigator (Prof Hiscock) will contact the caregiver to debrief and counsel as appropriate.
   5. Ask them if they would like CI Peyton to call an external agency on their behalf.

If the family declines further contact from the research team, it is important to respect their wishes, whilst ensuring that we do not ignore our legally mandated requirements for the child’s safety and welfare.

**Deterioration in child since referral**

If, during a discussion about the website for some reason a caregiver raises concerns regarding a deterioration of their child’s mental health or mental health treatment, CI Peyton will recommend the following available options to the family.

1. Discuss these concerns with their referrer (eg GP)
2. Discuss these concerns with the paediatrician, if available.
3. Discuss these concerns with national help lines (eg MCHN/Parentline) and clinical services as appropriate.

These issues will be followed up with Prof Hiscock. Prof Hiscock will then consider discussing these concerns directly with the family, and consider an appropriate clinical response, such as contacting the paediatrician who triaged the referral, and notify them of the parent’s concerns so they can consider an earlier review or other form of clinical support.

I will notify RCH HREC if the patient becomes distressed despite the initial procedure.

**Concern about child safety and welfare**

It is very unlikely that there will be a concern raised regarding the child safety or welfare, during the qualitative interview discussing their experience using the website. However, if a concern is raised regarding child protection notification, we are aware of our legal responsibilities, to which we are mandated as medical and psychology practitioners. In circumstances where we are concerned about the safety or welfare of the child, we may be legally required to report these concerns to the Department of Health and Human Services. This is part of Dr Peyton’s usual care, as a general paediatrician, and requires no change in practice. Dr Peyton has previously received formal training in child protection as part of his advanced training fellowship with the Royal Australasian College of Physicians. He has also conducted qualitative interviews with 16 parents previously (HREC 2019.160)

During the description of the research project, prior to consenting families, CI Peyton will use the telephone script to explain that all information is confidential, and won’t be disclosed without the participants permission, “except at required by law”.

If, during the participant interview, we become concerned about the child’s safety, we will:

- Explore the participant’s available avenues of support
- Let them know we are concerned about the situation
- Ask if they would be willing to contact additional help, or if we could contact that extra help on their behalf.
- Explain that, as a researcher, we are concerned for them and their child, and would like to talk to the supervisor of the research team (Professor Hiscock), to see if any additional support needs to be arranged.

Once the interview ends, CI Peyton will contact supervisor (Professor Hiscock) to discuss the interview, and our concerns, immediately. The supervisor will then decide on the best course of action, taking into account our legal and ethical obligations to the child. As a guide, we will consider our alternatives as:

- Notify child protection with concerns about children in need of protection
- Notify Child FIRST where there are concerns about a child’s wellbeing
- If appropriate, we can consult with RCH support services, such as Gatehouse, RCH social work department and RCH psychology services.

# Reporting of safety events

Although it is not expected that this intervention poses a risk of a serious adverse event, if there is a SAE, this is how it will be reported.

Any SAE occurring in a study participant will be reported to the HREC within 24-72 hours of occurrence, in accordance with the safety reporting policy of the HREC. The HREC safety reporting form will be completed, signed and submitted by an investigator.

# DATA AND INFORMATION MANAGEMENT

# Overview

CI Peyton is responsible for storing essential trial documents relevant to data management and maintaining a site-specific record of the location(s) of the site’s data management-related Essential Documents.

CI Peyton is responsible for maintaining adequate and accurate source documents. Source data will be attributable, legible (including any changes or corrections), contemporaneous, original, accurate, complete, consistent, enduring and available. Changes to source data (hardcopy and electronic) must be traceable, must not obscure the original entry, and must be explained where this is necessary.

CI Peyton will also maintain accurate case report forms (CRFs) (i.e. the data collection forms) and be responsible for ensuring that the collected and reported data is accurate, legible, complete, entered in a timely manner and enduring. To maintain the integrity of the data, any changes to data (hardcopy and electronic) must be traceable, must not obscure the original entry, and must be explained where this is necessary.

# DATA MANAGEMENT

# Data generation (source data)

In this trial, the following types of data will be collected:

- personal identifying information (names, dates of birth, contact details)
- sensitive information including health data (mental health symptoms, behaviour symptoms, health service use)

The source documents for this trial include:

- surveys completed by the participant through REDCap,
- signed verbal consent forms completed by the administration staff at the clinic and CI Peyton,
- anonymous website usage data from Google analytics,
- and audio recording and hand written notes written by CI Peyton during the semi-structured phone interviews.

# Data capture methods and data use, storage, access and disclosure during the trial

Data collection methods

Data for this trial will be collected and entered using electronic data collection forms which will be completed by the participant. Website usage data will be collected by Google analytics and manually transferred across to the relevant participant on REDCap, by CI Peyton. Google analytics collects anonymous website usage data on websites. Hardcopy field notes from the qualitative interviews will be collected by CI Peyton. The data will be used for the analyses specified in the protocol and Statistical Analysis Plan.

Following the completion and analysis of the trial, the data will be retained long-term following the mandatory archive period for use in future research projects (see section 9.2.5).

Storage and access

Hard copy data will be stored by the Site in a locked cabinet in a secure location, accessible to the research team only.

Electronic data will be securely stored in MCRI's REDCap database system and in files stored in MCRI's network file servers, which are backed up nightly. **Files containing private or confidential data will be stored only in locations accessible only by appropriate designated members of the research team.**

REDCap is hosted on MCRI infrastructure and is subject to the same security and backup regimen as other systems (e.g. the network file servers). Data is backed up nightly to a local backup server, with a monthly backup taken to tape and stored offsite. REDCap maintains an audit trail of data create/update/delete events that is accessible to project users who are granted permission to view it. Access to REDCap will be provided via an MCRI user account or (for external collaborators) via a REDCap user account created by the MCRI system administrator. The permissions granted to each user within each REDCap project will be controlled by, and will be the responsibility of, the trial team delegated this task by the Principal Investigator. REDCap has functionality that makes adding and removing users and managing user permissions straightforward. All data transmissions between users and the REDCap server are encrypted. The instructions for data entry to REDCap must be read and the training log signed prior to personnel commencing data entry on REDCap.

Authorised representatives of the sponsoring institution as well as representatives from the HREC, Research Governance Office and regulatory agencies may inspect all documents and records required to be maintained by the Investigator for the participants in this trial. The trialsite will permit access to such records.

Disclosure

The trial protocol, documentation, data and all other information generated will be held in strict confidence. No information concerning the trial or the data will be released to any unauthorised third party, without prior written approval of the sponsoring institution. Clinical information will not be released without written permission of the participant, except as necessary for monitoring by the HREC, Research Governance Office or regulatory agencies.

# Data confidentiality

Participant confidentiality is strictly held in trust by the research team. To preserve confidentiality and reduce the risk of identification during collection, analysis and storage of data and information, the following will be undertaken:

(1) The number of private/confidential variables collected for each individual has been minimised. The data collected will be limited to that required to address the primary and secondary objectives.

(2) Participant identifiers will be stored separately to the data collected; documents with identifiers will be stored separately to participant data. Participant data and samples will be identified through use of a unique participant trial number/code assigned to the trial participant (“re-identifiable”). The Site Principal Investigator is responsible for the storage of a master-file of names and other identifiable data with the participant ID; access to this document will be restricted to the site trial team and authorised persons as listed previously. The master file should be stored securely, and separately, from trial data in locked/ password-protected databases with passwords kept separately

(3) Separation of the roles responsible for management of identifiers and those responsible for analysing content. The data will be analysed by the statistician, who will be provided with anonymised data identified only by the unique participant trial ID.

# Quality assurance

CI Peyton will be responsible for data cleaning and site monitoring. Site monitoring will entail weekly checks with the clinic sites to ensure no problems with identification of eligible participants, recording these participants in the internal database, and recording parental consent to pass on contact details to CI Peyton.

# Record Retention

As per the Australian Code for the Responsible Conduct of Research, study records and data will be kept for a minimum of 5 years following the completion of the study. At 5 years, we will destroy the records, including audio recordings. Records will be destroyed by deleting the data from MCRI servers, and hard copies will be disposed of within confidential waste.

# TRIAL OVERSIGHT

# Governance structure

Governance of sites and the research project will be provided through two tiers:

- CI Peyton will meet with the supervisory team (Prof Hiscock, Dr Wadley and Dr Hackworth) every fortnight during the trial. These meeting will run for 1 hour. These meetings will be discuss all areas of the trial, from recruitment, timelines, consent and data collection, all matters relating to the intervention, data management and safety.
- CI Peyton will meet with the administration staff at each clinic on a weekly basis. These regular face to face meetings will allow any discussion about problems encountered in the recruitment phase of the trial.

Between these meetings, any urgent problems related to the trial can be addressed through direct communication between CI Peyton and supervisor Professor Hiscock.

# STATISTICAL METHODS

# Sample Size Estimation

As this is a pilot trial, there is no consistent agreement in the number of participants required to assess acceptability and feasibility. Typically, between 20-100 participants are recruited into similar trials assessing acceptability, feasibility and usability.^12,14,28^ As a pilot RCT, the trial is not powered to assess statistical significance of parent, child or health service-related outcomes.

The quantitative primary outcomes and secondary outcomes will be compared using descriptive statistical methods. Linear regression and logistic regression will be conducted to estimate mean differences (and 95% confidence intervals) for continuous outcomes, and odds ratios (and 95% confidence intervals) for binary outcomes, respectively, between trial arms. Analyses will be adjusted for baseline scores of the outcome measure. The research team will calculate Cohen’s d effect sizes for continuous outcomes to inform sample size calculations for the planned future RCT. The RCT will be reported in accordance with the CONSORT e-health statement and we will complete an intention-to-treat analysis at the level of the child.^29^

Qualitative interviews will be analysed using an inductive content analysis approach. This approach employs three main parts: i) open coding; ii) creating categories by cross referencing, and grouping the data; and, iii) abstraction.^30,31^ Overall, we hope to describe key issues, themes and events in the parent’s experience using the FindWays website.

# ETHICS AND DISSEMINATION

# Research Ethics Approval & Local Governance Authorisation

This protocol and the informed consent document and any subsequent amendments will be reviewed and approved by the human research ethics committee (HREC) prior to commencing the research. A letter of protocol approval by HREC will be obtained prior to the commencement of the trial, as well as approval for other trial documents requiring HREC review.

# Amendments to the protocol

This trial will be conducted in compliance with the current version of the protocol. Any change to the protocol document or Informed Consent Form that affects the scientific intent, trial design, participant safety, or may affect a participant’s willingness to continue participation in the trial is considered an amendment, and therefore will be written and filed as an amendment to this protocol and/or informed consent form. All such amendments will be submitted to the HREC, for approval prior to being implemented.

# Protocol Deviations and Serious Breaches

All protocol deviations will be recorded in the participant record in REDCap (source document) and must be reported to the supervisor Professor Hiscock, who will assess for seriousness.

Those deviations deemed to affect to a significant degree rights of a trial participant or the reliability and robustness of the data generated in the clinical trial will be reported as serious breaches. Reporting will be done in a timely manner (review and submit to the approving HREC within 7 days).

Where non-compliance significantly affects human participant protection or reliability of results, a root cause analysis will be undertaken, and a corrective and preventative action plan prepared.

Where protocol deviations or serious breaches identify protocol-related issues, the protocol will be reviewed and, where indicated, amended.

# CONFIDENTIALITY

Participant confidentiality is strictly held in trust by the participating investigators, research staff, and the sponsoring institution and their agents. This confidentiality is extended to cover the clinical information relating to participants.

The trial protocol, documentation, data and all other information generated will be held in strict confidence. No information concerning the trial or the data will be released to any unauthorised third party, without prior written approval of the sponsoring institution.

Clinical information will not be released without written permission of the participant, except as necessary for monitoring by HREC or regulatory agencies, or as required by law.

# PARTICIPANT REIMBURSEMENT

The participants will not receive reimbursement to participate in the study.

# FINANCIAL DISCLOSURE AND CONFLICTS OF INTEREST

The investigators declare no conflict of interest.

# DISSEMINATION AND TRANSLATION PLAN

CI Peyton holds the primary responsibility for publication of the results of the study. Upon completion of the study, results will be disseminated via four methods:

Publication of results in a peer-reviewed journal

Presentation of results to conferences

Presentation of results to local paediatricians in Geelong

Plain language summary or results to be distributed to parents agreeing to receive follow up results.

# REFERENCES

1. Lawrence D, Johnson S, Hafekost J, et al. *The Mental Health of Children and Adolescents. Report on the Second Australian Child and Adolescent Survey of Mental Health and Wellbeing*. Canberra: Department of Health; 2015.

2. Knapp, M.; McDaid, D.; Parsonage M. Mental Health Promotion and Prevention: The Economic Case. *UK Dep Heal*. 2011;(January):48. doi:10.15405/book.3.2

3. Stevenson J, Goodman R. Association between behaviour at age 3 years and adult criminality. *Br J Psychiatry*. 2001;179(SEPT.):197-202. doi:10.1192/bjp.179.3.197

4. Cunningham CE, Chen Y, Deal K, et al. The Interim Service Preferences of Parents Waiting for Children’s Mental Health Treatment: A Discrete Choice Conjoint Experiment. *J Abnorm Child Psychol*. 2013;41(6):865-877. doi:10.1007/s10802-013-9728-x

5. World Health Organisation. *WHO Guideline: Recommendations on Digital Interventions for Health System Strengthening*. Geneva; 2019.

6. Ryan GS, Haroon M, Melvin G. Evaluation of an educational website for parents of children with ADHD. *Int J Med Inform*. 2015;84(11):974-981. doi:10.1016/j.ijmedinf.2015.07.008

7. Deitz DK, Cook RF, Billings DW, Hendrickson A. Brief Report: A Web-Based Mental Health Program: Reaching Parents at Work. *J Pediatr Psychol*. 2009;34(5):488-494. doi:10.1093/jpepsy/jsn108

8. van Gemert-Pijnen JEWC, Nijland N, van Limburg M, et al. A holistic framework to improve the uptake and impact of eHealth technologies. *J Med Internet Res*. 2011;13(4). doi:10.2196/jmir.1672

9. Abras C, Maloney-Krichmar D, Preece J. User-centred design. In: Bainbridge WS, ed. *Berkshire Encyclopedia of Human-Computer Interaction*. Vol 1. Berkshire Publishing Group LLC; 2004.

10. Michie S, Yardley L, West R, Patrick K, Greaves F. Developing and evaluating digital interventions to promote behavior change in health and health care: Recommendations resulting from an international workshop. *J Med Internet Res*. 2017;19(6):1-19. doi:10.2196/jmir.7126

11. Enam A, Torres-Bonilla J, Eriksson H. Evidence-based evaluation of ehealth interventions: Systematic literature review. *J Med Internet Res*. 2018;20(11):1-18. doi:10.2196/10971

12. *Monitoring and Evaluating Digital Health Interventions: A Practical Guide to Conducting Research and Assessment*. Geneva: World Health Organization; 2016. http://apps.who.int/bookorders.

13. Epstein RM, Street RL. The values and value of patient-centered care. 2011.

14. Kauer SD, Buhagiar K, Blake V, Cotton S, Sanci L. Facilitating mental health help-seeking by young adults with a dedicated online program: a feasibility study of Link. *BMJ Open*. 2017;7(7).

15. Sanci L, Kauer S, Thuraisingam S, et al. Effectiveness of a mental health service navigation website (link) for young adults: randomized controlled trial. *JMIR Ment Heal*. 2019;6(10):e13189.

16. MCKAY M, Bannon WM. Engaging families in child mental health services. *Child Adolesc Psychiatr Clin N Am*. 2004;13(4):905-921. doi:10.1016/j.chc.2004.04.001

17. Goodman R, Meltzer H, Bailey V. The Strengths and Difficulties Questionnaire: a pilot study on the validity of the self-report version. *Eur Child Adolesc Psychiatry*. 1998;7(3):125-130.

18. Stone LL, Janssens JMAM, Vermulst AA, Van Der Maten M, Engels RCME, Otten R. The Strengths and Difficulties Questionnaire: psychometric properties of the parent and teacher version in children aged 4–7. *BMC Psychol*. 2015;3(1):1-12.

19. Hawes DJ, Dadds MR. Australian data and psychometric properties of the Strengths and Difficulties Questionnaire. *Aust N Z J Psychiatry*. 2004;38(8):644-651. doi:10.1111/j.1440-1614.2004.01427.x

20. Goodman R. The extended version of the Strengths and Difficulties Questionnaire as a guide to child psychiatric caseness and consequent burden. *J child Psychol psychiatry*. 1999;40(5):791-799.

21. Antony MM, Bieling PJ, Cox BJ, Enns MW, Swinson RP. Psychometric properties of the 42-item and 21-item versions of the Depression Anxiety Stress Scales in clinical groups and a community sample. *Psychol Assess*. 1998;10(2):176.

22. Crawford J, Cayley C, Lovibond PF, Wilson PH, Hartley C. Percentile norms and accompanying interval estimates from an Australian general adult population sample for self‐report mood scales (BAI, BDI, CRSD, CES‐D, DASS, DASS‐21, STAI‐X, STAI‐Y, SRDS, and SRAS). *Aust Psychol*. 2011;46(1):3-14.

23. Moore G, Wilding H, Gray K, Castle D. Participatory methods to engage health service users in the development of electronic health resources: Systematic review. *J Particip Med*. 2019;11(1). doi:10.2196/11474

24. Fogg BJ. Persuasive technology: using computers to change what we think and do. *Ubiquity*. 2002;2002(December):5.

25. Kauer S, Buhagiar K, Sanci L. Facilitating mental health help seeking in young adults: the underlying theory and development of an online navigation tool. *Adv Ment Heal*. 2016;15(1):71-87. doi:10.1080/18387357.2016.1237856

26. Algate F, Gallagher R, Nguyen S, Ruda S, Sanders M. EAST.

27. Broglio K. Randomization in clinical trials: permuted blocks and stratification. *Jama*. 2018;319(21):2223-2224.

28. Hiscock H, Ng O, Crossley L, Chow J, Rausa V, Hearps S. Sleep Well Be Well: Pilot of a digital intervention to improve child behavioural sleep problems. *J Paediatr Child Health*. 2021;57(1):33-40.

29. Eysenbach G, CONSORT-EHEALTH Group. CONSORT-EHEALTH: improving and standardizing evaluation reports of Web-based and mobile health interventions. *J Med Internet Res*. 2011;13(4). doi:10.2196/jmir.1923

30. Elo S, Kyngäs H. The qualitative content analysis process. *J Adv Nurs*. 2008;62(1):107-115. doi:10.1111/j.1365-2648.2007.04569.x

31. Braun V, Clarke V. Using thematic analysis in psychology. *Qual Res Psychol*. 2006;3(2):77-101.

# Appendix 1: Division of sponsor responsibilities between sponsor and sponsor-investigator

| **Sponsor-Investigator:** | *Daniel Peyton* | | |
| --- | --- | --- | --- |
| **Responsibility** | | **Sponsor** | **Sponsor-Investigator** |
| Ensure a peer review/independent expert review has demonstrated that the trial proposal is worthwhile and is of high scientific quality. | | X |  |
| Ensure the Sponsor-Investigator has adequate procedures in place for all key trial management activities | | X |  |
| Assign an overall risk category based on type of intervention | | X |  |
| Ensure that the Sponsor-Investigator has the necessary expertise and experience to conduct the trial | | X |  |
| Ensure that the Sponsor-Investigator has the resources needed to complete the trial successfully or that plans are in place to raise additional funds. | | X |  |
| Confirm provision of insurance and indemnity for the trial and trial related staff as well as measures for participant compensation for trial related injury | | X |  |
| Ensure all the roles and responsibilities for the clinical trial are delegated, agreed and documented appropriately | | X |  |
| Oversee/sign-off all contract negotiations with external providers (e.g. external lab facilities; pharmaceutical companies for supply of investigational product,) | | X |  |
| Ensure the protocol (or other document) details appropriate monitoring and management plans commensurate to the risk and complexity of the trial | | X |  |
| Maintain oversight to include audit, where applicable | | X |  |
| Ensure that the trial is based on a thorough review of scientific literature including whether any relevant systematic review exists. | |  | X |
| Secure funding and/or confirm sufficient resources are available to conduct the trial (e.g. trial subjects, time, staff, facilities, finances) or put in place plans to raise additional funds. | |  | X |
| Ensure that trials are registered on clinical.trials.gov, ANZCTR or other appropriate registry before first patient is enrolled and that appropriate plans for the dissemination of trial findings are in place | |  | X |
| Unless delegated to a third party, undertake/oversee the design, conduct and reporting of the trial with support from all relevant specialist staff (e.g. statistician, research methodologist) including the development of a protocol that is compliant with international standards including the [SPIRIT Statement](http://www.spirit-statement.org/). | |  | X |
| Ensure a trial risk assessment has been carried out and proportionate trial management and monitoring plans are in place | |  | X |
| Develop/endorse an appropriate strategy for independent trial oversight (e.g. Trial Management Group, Trial Steering Committee, Data Safety Monitoring Board)  If a Data Safety Monitoring Board is not warranted, ensure alternative mechanisms for ongoing safety monitoring are in place | |  | X |
| Document trial specific delegation of duty on a Staff Signature and Delegation Log | |  | X |
| Confirm each member of the trial team are aware of their trial-related duties | |  | X |
| Ensure the development of all relevant trial documentation (e.g. protocol, Participant Information and Consent Form and Case Report Form) | |  | X |
| Clearly identify the reference safety information to allow identification of expectedness of adverse events | |  | X |
| Oversee the set-up of a clinical trial database | |  | X |
| Ensure all trial approvals and notification are in place before the trial commences (e.g. HREC, SSA, TGA) | |  | X |
| Ensure relevant agreements/signatories from service departments supporting the trial (e.g. laboratories, radiology) are obtained | |  | X |
| Ensure arrangements are in place for the effective financial management of the trial | |  | x |
| Prepare and submit amendments to the trial | |  | X |
| Implement procedures to ensure the collection of high quality and accurate data | |  | X |
| Oversee the set-up and maintenance of a Trial Master File | |  | X |
| Ensure safety reporting and monitoring for the trial complies with the requirements of the NHMRC Guidance for Safety Monitoring and confirm and execute any sponsor reporting responsibilities that are delegated | |  | X |
| Submit annual report(s) to the HREC and Research Office in accordance with Australian Guidance and local requirements | |  | X |
| Report suspected serious breaches of GCP/protocol to the HREC and Research Office in accordance with the NHMRC Guidance | |  | X |
| Notify HREC, Research Office and other relevant bodies of the completion of the trial | |  | X |
| Produce all necessary reports to funders and others | |  | X |
| Disseminate trial findings through publication/dissemination of trial results where applicable, following the CONSORT Statement | |  | X |
| Fulfil commitments to trial participants, such as providing information about the outcome(s) of the trial, re-obtaining consent if required due to change in risk-benefit ratio (of investigational medicinal product) or change in protocol procedures | |  | X |
| Ensure all trial data (including the Trial Master File) and materials, are archived appropriately and retrievable for audit purposes | |  | X |
| Maintain trial registration record in accordance with the registry’s requirements | |  | X |
